# Acupuncture Case Registry Study: Rationale, Implementations, and Achievements

**DOI:** 10.1155/2022/3860231

**Published:** 2022-11-21

**Authors:** Tianyi Zhao, Jia Liu, Yiming Li, Hongjiao Li, Chao Wang, Zhuoxin Yang, Xin Wang, Yanke Ai, Yuning Qin, Xue Cao, Lihong Yue, Zhishan Ge, Shihua Wang, Xiangran Meng, Yanjun Wang, Liyun He

**Affiliations:** ^1^Institute of Basic Research in Clinical Medicine, China Academy of Chinese Medical Sciences, Beijing, China; ^2^Swiviss University of Traditional Chinese Medicine, Hauptstrasse 61, Bad Zurzach 5330, Switzerland; ^3^Department of Acupuncture and Moxibustion, Shenzhen Traditional Chinese Medicine Hospital, Futian District, Shenzhen, China; ^4^China Academy of Chinese Medical Sciences, Beijing, China; ^5^Department of Acupuncture and Moxibustion, The First Affiliated Hospital of Hebei University of Chinese Medicine, Chang'an District, Shijiazhuang, Hebei, China

## Abstract

The acupuncture case registry study is focusing on acupuncture therapy data from patient cases. The main objective is to collect real-world data and integrate clinically meaningful outcome evaluation indicators to uncover and evaluate real-world acupuncture efficacy and safety, explore factors affecting acupuncture efficacy, and provide real-world evidence to complement RCTs. Since the International Acupuncture Case Registry data collection system's establishment in 2017, 16 projects have been underway, including two acupuncture specialty therapies and 15 diseases. Data from 3404 patients included extensive information on the diagnosis and treatment of acupuncture and the evaluation of its efficacy. In order to serve as a guide for future studies, this article discusses the value of and rationale for establishing acupuncture case registry studies, how to distinguish them from patient registries, and crucial techniques for implementing registry studies in terms of applications, patient recruitment, costakeholder collaboration, data collection and management, study quality control, and ethics.

## 1. Introduction

Acupuncture is a technique that is used to stimulate specific body points, usually by inserting fine needles into the skin. Acupuncture has been used in China for 2,500 years and is increasingly used to treat various conditions. The World Health Organization 2019 Global Report [1] revealed that acupuncture is widely used in 183 countries and regions worldwide for 461 symptoms and 972 diseases [2], and some countries have incorporated it into their health insurance and medical regulations. More than 10 million acupuncture treatments are performed annually in the United States [3], and the National Health Service provides over four million acupuncture treatments [4]. An increasing number of randomized controlled trials (RCTs) have been conducted in recent decades in terms of clinical research to study the efficacy and safety of acupuncture. More clinical studies of acupuncture, especially RCTs have increased as evidence-based medicine continuously develops. In the last 10 years, seven acupuncture clinical trial publications have been published in the world's top medical journals [5], including the Annals of Internal Medicine [6] and the Journal of the American Medical Association [7]. These articles document the clinical effectiveness of acupuncture and the quality of clinical studies in China. However, RCTs are limited in themselves and require strict implementation of randomization and blinding principles. RCTs cannot fully meet the needs of clinical evidence generation in traditional Chinese medicine (TCM). Highlighting the strengths and characteristics of TCM is difficult, resulting in acupuncture therapies that are being determined clinically effective showing negative results in most RCTs.

Therefore, further development of real-world studies of acupuncture is necessary. Rather than operating in a “sterile” environment with a narrowly defined audience, the real-world study assesses the effectiveness and safety of an intervention with a more diverse audience in a real-world setting. Therefore, real-world studies could provide a valid and valuable method for acupuncture assessment [8]. Conducting real-world studies of acupuncture to complement the evidence from RCTs of acupuncture is important.

Registries for evaluating patient outcomes and the real-world evidence they provide are highly valuable to physicians and patients. Registries increase the understanding of treatment effects and the natural history and progression of the disease and allow the observation of large patient populations [9].

Therefore, Prof. Liu, who is the principal investigator of our team, proposed the idea and methodology of an international acupuncture registry study in 2013 [10]. By further design, the first international acupuncture case registry study started in science in 2017, which aimed to establish a worldwide networked data platform for evaluating the clinical efficacy and safety of acupuncture treatments [11].

Registry studies were not clearly classified, and the common names currently used are “patient registry” and “case registry.” The term “patient” shows that the registry primarily concerns health information. It is a broad term that can include people with a certain disease, pregnant or lactating women, or people who have a birth defect or a molecular or genomic feature [12]. The term “case” focuses on collecting data on specific eligible illness cases. The “Case Registry” studies are more concerned with public health surveillance or the tracking and documentation of data from studies of eligible patients [13, 14]. Based on our review of previous registry studies and real-world studies of acupuncture, the acupuncture case registry study differs from most existing patient registry studies for a particular disease.  Table 1 shows the comparison of the specific differences. The acupuncture patient registry studies primarily aimed to evaluate the clinical efficacy of acupuncture rather than to epidemiologically investigate diseases. Based on this research purpose, the design of the study should reflect the diagnostic and therapeutic characteristics of acupuncture, such as the registration and analysis of acupuncture operational details (e. g., acupuncture site/acupoints, techniques, and the state of the doctor and patient) and acupuncture diagnostic and therapeutic thinking (e. g., identification/diagnosis).

This manuscript outlines the use of key techniques and specific considerations for the implementation and application of acupuncture patient registries.

## 2. Methods and Implementations

### 2.1. Objectives and Applications

The acupuncture case registry is a study focusing on acupuncture therapy data from patient cases, making it primarily distinct from a patient registry which is a study designed to collect epidemiological data on the disease [16].  Table 1 shows the difference between the acupuncture case registry and patient registry in terms of study purpose. The acupuncture case registry has a more specific purpose, with efforts focused on collecting data on how acupuncture is used in the clinic and tracking the ongoing impact of acupuncture on patients to uncover the diagnostic and therapeutic patterns of acupuncture. This is different from the primary purpose of a patient registry, which is to describe the natural history of the disease.

Acupuncture registry studies can be conducted for the following applications.

#### 2.1.1. Evaluating the Clinical Efficacy and Cost-Effectiveness of Acupuncture

In contrast to RCTs that require strict inclusion and exclusion criteria, patient registration studies have a relatively broad population, making them suitable for examining the efficacy rather than the efficacy of acupuncture in actual clinical practice. Concurrently, the registration mechanism is more convenient for the study population. Furthermore, the registration mechanism is more suitable for the long-termfollow-up of the study population, long-term efficacy observation of acupuncture, and dynamic change analyses in the treatment process of acupuncture, as well as flexibility in adjusting the study protocol according to the current situation during the study and providing more comprehensive data for efficacy evaluation or related critical factors exploration. Registration mechanisms can be used to evaluate the cost-effectiveness of acupuncture in the real-world. Cost-effectiveness is a way of describing the relative value of the ability of a healthcare product or service to achieve the desired outcome for the given resources spent, thereby comparing costs with measured clinical regression [17]. Cost-effectiveness models are developed to assess the cost-effectiveness of the procedure by collecting data related to the costs and benefits of acupuncture or by linking it to health insurance data during acupuncture case registry studies.

#### 2.1.2. Description of the Acupuncture Application

Describing acupuncture applications in different regions and countries provides a clear picture of the theoretical foundation, methods, prescriptions, acupuncture point selection, techniques, and other essential acupuncture applications in clinical practice. Additionally, it discusses the types of diseases that can be treated by acupuncture, the population that receives acupuncture treatment, and the differences in the basic situation of acupuncture in different regions and countries. Until now, no one has studied and analyzed these basic acupuncture applications. A descriptive study that uses data from a case registry could answer this question. These works can provide preliminary studies to explore the spectrum of acupuncture diseases, dominant diseases, and treatment norms.

#### 2.1.3. Monitoring and Documentation of Acupuncture Safety

Acupuncturists can promptly record adverse events during treatment [18], such as dizziness, needle breakage, hematoma, etc., in a patient case registry study, and disease-related adverse reactions should be recorded to observe whether acupuncture has reduced the occurrence of disease-related adverse reactions compared to other treatment methods. Specific advantages include: first, increased motivation of physicians to record adverse events and focused follow-up of patients with adverse events, especially for the recording of minor symptoms or easily-ignored acupuncture adverse events, such as bleeding after acupuncture and thick needles; second, the existing electronic medical record lacks records of adverse acupuncture events or has incomplete records and cannot accurately estimate the incidence of adverse events in the total population. The case registry addresses this issue by improving the reporting of the incidence of adverse acupuncture events.

#### 2.1.4. Evaluation of Clinical Treatment Quality and Patient Satisfaction in Acupuncture

Acupuncture's clinical efficacy is influenced by a variety of factors, including the acupuncturist's experience and technical mastery of the consultation and treatment, as well as nonspecific factors like the patient's psychological expectations before acupuncture treatment [19], satisfaction after acupuncture, the patient's psychological treatment environment state [20], comfort while receiving treatment, and the acupuncturist's experience and technical mastery of the consultation and treatment. The consistency of these health services with available expertise and the amount to which individual and population health services increase the chance of expected health regression are used to determine the quality of care. Case registry studies can indirectly aid in improving clinical outcomes and better individualizing acupuncture treatment by including a patient satisfaction survey module, combining data on follow-up rates and the number of follow-up visits, and comparing differences in the quality of care between physicians or providers.

In summary, the most important thing for acupuncture case registry studies at the project stage is to clarify the clinical significance of the project, prioritize clinical studies of acupuncture advantageous diseases or potential advantageous diseases, as well as acupuncture characteristic techniques to conduct registry studies to support clinical evidence from acupuncture RCTs, expand the scope of acupuncture clinical efficacy evaluation studies, and enhance the quality of acupuncture clinical efficacy evaluation studies [8].

### 2.2. Patient Recruitment and Costakeholder Collaboration in Study Implementation

Personal health information and patient-generated data are becoming increasingly available as vast amounts of data and real-world evidence emerge. Increasingly, individuals and community organizations are seen as research partners as shared decision-making increases, particularly in the design and management of studies involving patient registries [21]. Therefore, the collected data will be more extensive and more authentic if we can consider the enthusiasm of primary care institutions and individuals for registration, and more meaningful results can be produced.

The study implementation should start with the participation of medical institutions (hospitals, associations, etc.) and therefore should include researchers, clinicians, service providers in their industry, and relevant community managers, all of whom can participate in the collaborative study [22]. In an acupuncture case registry study, physician recruitment should be conducted on the platform of a medical institution. The investigator needs to fully consider the international and domestic clinical status of acupuncture, screen the study institution, recruit acupuncturists to participate through multiple channels, and recruit patients through physicians. Additionally, achieving research objectives and participating in acupuncture case registry studies can improve physicians' clinical research skills, standardize acupuncture clinical practice, increase interest in clinical research, increase patient-physician interaction, facilitate the promotion and introduction of acupuncture therapies, and promote acupuncture culture. Finally, a physician recruits patients who participate in the study. The project needs to be centered on a clear research goal at the research initiation stage, with the interests of multiple participants identified and prioritized, to better conduct the work and maintain long-term, stable research [23]. It also needs to assess the conflicts of interest among participants that affect the bias of the study results and try to avoid such conflicts.

Moreover, we attempted to establish an academic community of acupuncture for POI by establishing acupuncture expert studios and professional academic committees in the implementation of the registry research of acupuncture for premature ovarian insufficiency (POI). Nearly 30 acupuncture POI expert workshops have now been established in China. The World Federation of Acupuncture-Moxibustion Societies and the China Association for Acupuncture and Moxibustion have taken the lead in forming an expert committee, which has included experts in acupuncture, evidence-based medicine, clinical epidemiology, statistics, obstetrics and gynecology, and other fields, to discuss acupuncture intervention protocols, efficacy evaluation methods, data management, quality control, and other topics. Experts worldwide attended the meeting. The cross-discipline exchange and learning of these experts improve the quality of case registry studies and deepen clinicians' disease knowledge, which not only ensures the smooth operation of registry studies but also aids clinicians in improving diagnosis and achieving early detection and treatment of POI [11].

### 2.3. Data Collection and Data Stewardship

The “Framework for FDA's Real-World Evidence Program” recognizes the importance of patient registries as a source of real-world data and emphasizes processes to minimize missing or incomplete data, as well as the collection of rigorous and high-quality data, thereby ensuring that the generated data can be used as evidence in the real world [24]. Fundamental, structural, semantic, and organizational interoperability techniques must be created and widely embraced, following specific sections of the 21^st^ Century Cures Act, to improve current research and development. The primary and secondary data sources for the registry should be defined because this is a real-world clinical trial. Secondary data is received through external links to electronic medical record systems or medical data systems of other institutions, and primary data is collected on a standardized data platform. Technical steps are adopted to ensure the feasibility of data linkage and thorough quality control of secondary data to limit the dangers of data linkage, assure the accuracy of data, and protect patient privacy [25]. Data was gathered in addition to documenting the general features of the patients and their medical problem information. The researcher should focus on refining the collection of acupuncture-related data when designing the registration form, primarily including (1) information on clinical acupuncture prescription and clinical acupuncture operation should be described in detail, which can be clearly displayed and illustrated using text, pictures, or videos; (2) acupuncture operation information, such as acupuncture point selection, acupuncture tool selection, body position, tonic and diarrheal techniques, stabbing methods, auxiliary acupuncture techniques, needle holding methods, needle entry methods, needle angles, needle directions, needle depths, acupuncture techniques, acupuncture techniques, acupuncture duration, needle retention methods, needle retention times, and records of changes in the method of starting acupuncture; (3) influencing factors on the clinical effect of acupuncture, such as such as the psychological state of the acupuncturist and the patient during the treatment period and the characteristics of the acupuncturist (age, years of practice, and seniority). Furthermore, based on a case registry study of chronic low back pain treated via acupuncture, we found that (1) it is difficult for patients to be visited on the planned data collection date, so key evaluation points should be pre-determined during protocol design; (2) compared with patient registry studies, acupuncture case registry studies have shorter observation periods; thus, establishing enough follow-up duration to see the long-term advantages of acupuncture is necessary; (3) Some disease characteristics, such as pain quality, causes of pain aggravation and relief, and pain provoke, are not critical influencing factors in the analysis of the efficacy of acupuncture for low back pain and are nonmandatory data collection, so ignoring the collection of these data can reduce the researcher's workload [26].

We advocate putting in place a more complex data management system before collecting patient data in terms of data governance to ensure that the collected information meets the basic standards and best practices for clinical inquiries. Establishing a data quality assurance program to monitor and manage data attributability, readability, simultaneity, originality, regularity, consistency, completeness, timeliness, accuracy, authenticity, persistence, and accessibility are among the specific needs [27]. A strict data management plan is in place to ensure data quality, with data managers tracking and spot-checking the data regularly, providing timely feedback on quality issues with the entered data, and encouraging researchers to correct them as soon as possible with in-platform alerts and e-mail reminders. Statisticians were involved, and suspect data is constantly queried and processed [28]. The above data spot-checking, query issuing, and response must be completed and recorded within the registration platform.

Our research team has created the International Acupuncture Case Registry Platform for Real-World Study, which is based on the Chinese Academy of Traditional Chinese Medicine's “Research Integration Platform (https://www.amreg.org/).” The platform supports programs that recruit volunteer acupuncturists through the World Federation of Acupuncture-Moxibustion Societies Research Center and records patients who have received acupuncture therapy in the system, with the cooperation of 189 member organizations in 53 countries. This system primarily performs data collection and operation administration. The acupuncture case registry database built on this platform will become a tremendous resource for acupuncturists worldwide with the long-term development of this study, thereby giving comprehensive treatment data. Physicians will be able to use these tools for free to solve clinical problems connected to acupuncture and enhance clinical outcomes, with the permission of the study's expert committees [10].

### 2.4. Quality Control and Ethics

Acupuncture case registry studies are “multicenter, large sample, and diversified treatment” in nature, which require strong quality control (QC) to assure their scientific validity [29]. Real-time data quality monitoring is necessary to ensure the smooth implementation of high-quality registries because of the large sample size, wide geographic distribution, and long follow-up period [30]. This also determines whether the collected dataset meets the purpose of the study and is of high analytical value [31]. Thus, QC efforts need to be designed from the start of the study design and throughout the study process to ensure that this principle is followed. We have developed a combined remote and on-site quality control methodology during the last 5 years [32], with remote QC as the primary focus and on-site QC as a supplement.

This registration platform was used to undertake remote QC. Each clinical center was expected to conduct online QC by a data monitor for every 2-3 new cases entered during the study period to ensure the quality of subsequent cases. The monitors were then asked to log in to the platform once a week to check the number of cases and to undertake online monitoring every 10–15 cases to provide prompt feedback and recall issues. Online platform QC, telephone interviews, e-mail call-backs, and social networking software call-backs are all examples of remote QC approaches. Online data QC is only one component of the study because the acupuncture case registry study primarily focuses on evaluating an intervention. QC efforts at the study site are also necessary to ensure that acupuncture, as a treatment method, fits specifications during the study and determine the investigator's proficiency in mastering the acupuncture treatment protocol and the completeness of the operational information recorded. We adopted an audit system of first-level inspection by participating hospitals, second-level monitoring by the lead unit, and third-level supervision by high-level experts to conduct regular audits [33]. We conducted regular monitoring mainly on study data management, physician compliance with the study protocol, patient compliance, acupuncture operation accuracy, and the completeness of registration of acupuncture-related information to ensure a high-level implementation of study procedures.

A case registry must be designed, implemented, and maintained following ethical, legal, social, and privacy considerations for it to be successful [22]. An Institutional Review Board is an independent ethics committee that oversees research studies and ensures that the protocol, governance, protections, and methods are ethical and appropriate [22]. It is required for any study that collects identifiable information from human subjects. In all cases, study participation is entirely voluntary and elective, and participants are free to withdraw at any moment. Participants or their legally authorized representatives must provide informed consent to the collection, storage, and use of their health data once they have been enrolled before sharing any personal data with third parties [34]. They must be evaluated and authorized by an independent ethical committee before starting any acupuncture case registry studies. Multicenter studies should be ethically approved at each participating institution, and each registered patient should be informed, explained, and asked to sign an informed consent form before participating. We have implemented an electronic informed consent feature on our data platform to ensure that patients obtain proper informed consent and to reduce the occurrence of falsely informed circumstances. The patient can request a video call with his or her physician through the internet after completing a question-and-answer session about the study design, process, and patient rights relating to the study. Upon deciding to join, the patient will manually check off several conditions that demonstrate informed consent, such as affirmation that they have read and understood the study information and consent form, as well as certification that any questions they had were well answered. Patients who answered “yes” to all questions will be required to electronically sign and be formally enrolled in the study. The platform is also developed with the need for health information confidentiality in mind, employing technical measures and permission settings to actively preserve patient privacy information, such as eliminating identifiable patient information, from the database [35].

## 3. Achievements

One of the most important sources of evidence for acupuncture clinical research will be the creation of an international acupuncture case registry research platform and the development of case registry research with acupuncture features. The Acupuncture Case Registry Research Consortium of the China Association for Acupuncture and Moxibustion was officially created on February 11, 2017 [35], and the first initiatives include patient registry research on premature ovarian failure, chronic low back pain, and floating needle therapy. Therefore, the first international real-world acupuncture study was formally formed, with an expert steering committee and an acupuncture patient registration center run by the Clinical Evaluation Center of the Chinese Academy of Traditional Chinese Medicine's Institute of Clinical Basic Medicine.

**Figure 1:**
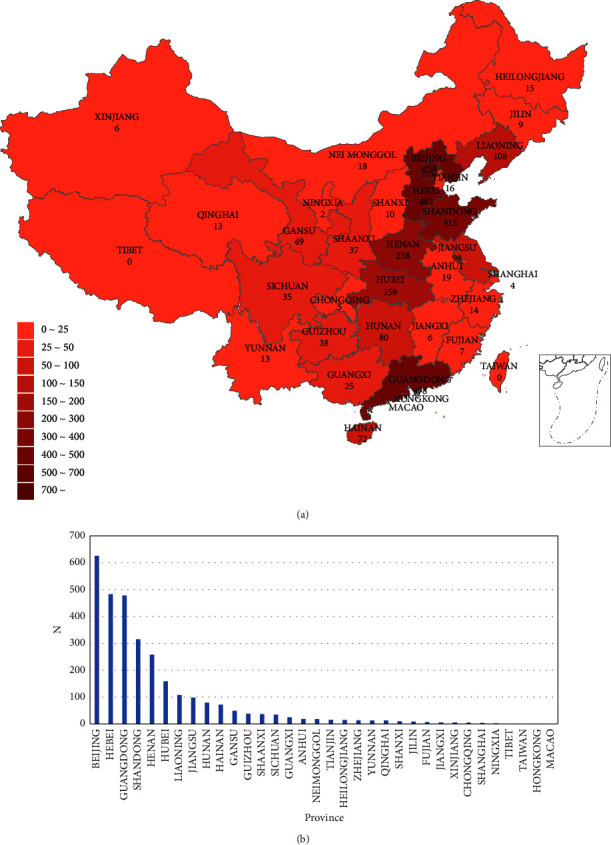
Key facts about the acupuncture patient registry. (a) The global reach of acupuncture patient registry. Provinces with active acupuncture patients are indicated in red. (b) Ranking chart of the number of registered cases by province.

The acupuncture case registry project is operational in many provinces around China. The registry has significantly grown over the past 9 years, and 3404 patients from 16 studies in 30 provinces have been enrolled as of March 30, 2022 (Figure 1). This study has continued to grow since its initiation and is currently the largest international study of acupuncture patient registries.  Table 2 demonstrates the 16 individual research projects that are currently in progress, including two studies of specialty acupuncture techniques, involving 15 diseases. Additionally, the studies now have four officially published research papers. Two-phase analyses have been published since the beginning of acupuncture studies for POI, which examine the differences in the effects of acupuncture with various stimulation intensities on increased sinus follicle counts [36] and the development of a columnar line graph clinical prediction model of the effects of acupuncture on pregnancy outcomes in patients with early-onset ovarian insufficiency (POI) [37]. This indicates that this study is in the phase of clinical data accumulation, and we estimate a successive research output in the last few years.

We presided over the drafting of the Standard for Management of Registry Study on Acupuncture-Moxibustion to better conduct international acupuncture patient registration, and the consultation draft of this specification was made public in the CAAM (https://www.ttbz.org.cn/Home/Show/21183) on January 13, 2021.

## 4. Looking to the Future

Since their inception, acupuncture case registry studies have significantly contributed to the advancement of clinical acupuncture-moxibustion research. The acupuncture case registry study preserves the individualized characteristics of the syndrome differentiation and acupuncture treatment. Additionally, the open-ended inclusion and exclusion criteria enable greater generalisability of the results of a study. As a result, it will play a significant role in the structure of evidence-based medicine for acupuncture. As the scope of the acupuncture case registry study expands, the Research Integration Platform will need to be modified to ensure the most relevant data are captured. In the future, new technologies may allow for faster and more efficient data aggregation from electronic medical records as well as other sources, such as smartphone app-based patient-reported outcomes or mobile data-logging devices.

In addition, future studies need to consider the registry of individuals receiving long-term acupuncture treatment. Tracing individual patients who receive acupuncture treatment for different reasons is necessary. This is not a traditional medical record data collection, and we will attempt to create a specifically relevant registry study to explore the role of acupuncture in public health.

There is no doubt that case registry studies can be applied to other forms of traditional Chinese medicine (TCM). There are already case registry studies of proprietary Chinese medicines, such as the case registry study of Nao Shuan Tong capsules for the treatment of ischemic stroke based on the theory of “toxic damage to brain collaterals,” which began in 2019 and has collected over 5,000 cases (https://www.news.cn/video/2022-08/16/c_1211676629.htm); and the prospective case registry study of Jianpi Sheng Xue tablets/granules for the treatment of iron deficiency anemia during pregnancy, which was conducted in 2017-2018 and included a total of 10,000 patients [39]. Both studies focused on the beneficial population as well as the clinical action features of proprietary Chinese medicines, which can be valuable for clarifying the scope of action and increasing clinical efficacy. In general, the current TCM real-world case registry studies are in their infancy. Our team intends to expand on the experience and platform of the acupuncture registry study to further the Chinese medicine case registry study. Due to the complex characteristics of syndrome differentiation and TCM treatment, collecting data can be challenging. Thus it is feasible to begin the case registry study using proprietary Chinese medicine or Chinese medicine injections [40]. Soon, with the improvement of TCM case registry study methodology and technology maturity and the development of a professional team, it will be possible to conduct a case registry study of Chinese herbal prescriptions in order to collect comprehensive data on TCM characteristics and investigate the clinical benefits of TCM treatments.

## 5. Conclusions

Acupuncture case registry studies were conducted for >5 years, and the currently collected information on acupuncture interventions and patients treated with acupuncture is greater and more extensive than any other previous clinical study. Our study develops a discussion of the purpose of the acupuncture patient registry, the three aspects of data collection and management, the discussion of common stakeholders in our implementation, and the differences from existing patient-centered registry studies to maximize the clinical value of the study and its value to the clinical development of acupuncture. Finally, this study will help ensure the acupuncture case registry study's understanding, improve the conducted follow-up quality, increase the active participation of the investigators in this study, and facilitate our management of this study.

## Figures and Tables

**Table 1:** Differences between a acupuncture case registry study and a patient registry.

Topics	Acupuncture case registry study	Patient registry [15]
Definition	Prospective observational studies that use acupuncture as the primary intervention and collect data uniformly to evaluate clinical efficacy and cost-effectiveness, describe the application of acupuncture therapy, monitor the safety of acupuncture therapy, evaluate the quality of acupuncturists, or conduct epidemiological disease investigation.	Organized system that collects uniform data (clinical and other) to identify specified outcomes for a population defined by a particular disease, condition, or exposure.
Objective	Primary purposes include assessing the clinical efficacy and cost-effectiveness of acupuncture, describing the application of acupuncture techniques, monitoring treatment safety, and evaluating the quality of acupuncturists.	Registry purposes can be broadly described in terms of patient outcomes. Major purposes include describing the natural history of disease, determining clinical and/or cost-effectiveness, and assessing safety or harm.
Patient enrollment	As determined by the study objectives, enrolled patients with a disease/certain symptom or a condition who have had acupuncture therapy from an acupuncturist or other acupoint stimulation interventions; generalisability of the acupuncture data and study results to be documented.	Aimed at enrollment of all patients with a disease or condition; generalisability of registry data to be documented.
Follow-up	Timelines are determined by the specific study objectives as well as the collection/extraction and processing of important study data.	Schedules drive timeliness for data collection and any anticipated data analyses which prompted the registry.
Data collection	Data is collected based on the objective of the registry study. A core set of data elements to be collected is agreed upon, and their definitions, coding systems, and data entry procedures are written down. Data collected for the purpose of a registry study can involve the primary collection of data or the secondary use of data.	
Data quality management	Study-specific data quality management will be defined in advance and executed using a risk-based approach, with a focus on regular surveillance and inspection of registered data.	Quality management is applied consistently to data and processes, with an emphasis on a core set of data elements; data quality management is specified and documented in advance.
	Data management systems to ensure data integrity, completeness, and security	
Analysis plan	Most of the time, detailed statistical considerations are written in a document separate from the study and registry protocols. This document is called a descriptive or hypothesis-driven statistical analysis plan.	Plan for statistical analysis, with analyses often done regularly at regular intervals based on patient accrual or analyses of predefined outcomes at time points described in the registry protocol.

**Table 2:** Acupuncture case registry research project and data collection^*∗*^

Acupuncture therapy	Patients	First patient enrolled	Cases	Published articles
Acupotomy	Lateral epicondylitis	2022.03	3	—
Acupotomy	Subacromial impingement syndrome	2022.03	0	—
Acupotomy	Upper limb spasticity after stroke.	2022.03	4	—
Acupotomy	Calcaneodynia	2022.03	0	—
Acupotomy	Knee osteoarthritis	2022.03	2	—
Acupotomy	Tenovaginitis of flexor digitorum	2022.03	0	—
Acupuncture therapy, not limited to specific types	Premature ovarian insufficiency	2017.06	1319	Clinical prediction model establishment of the influence of acupuncture-moxibustion on pregnancy outcome of patients with premature ovarian insufficiency based on patient registry study and LASSO-Cox model [36]. Effects of acupuncture on antral follicle count in patients with premature ovarian insufficiency based on patient registry study [37]
Acupuncture therapy	Postpartum depression	2018.09	433	—
Acupuncture therapy	Neurogenic urinary retention	2019.08	152	—
Custom blood therapy for acupoints	Urticaria chronica	2019.01	501	—
Acupuncture therapy	Asthenic bulbar paralysis	2022.03	6	—
Acupuncture therapy	Chronic low back pain	2017.06	573	Acupuncture therapy for chronic low back pain: protocol of a prospective, multicenter, registry study [38]
Acupuncture therapy	Prediabetes	2018.03	104	—
Acupuncture therapy	Degenerative osteoarthritis	2018.05	309	—
Acupuncture therapy	Diabetes prevention	2018.03	0	—
Acupuncture therapy	Malignant pleural effusion	2019.04	5	Discussion on registry study of acupuncture-moxibustion for malignant pleural effusion

^*∗*^The data in the table are up to 2022.3.30.

## Data Availability

No data were used to support this study.

## Abbreviations

QC: Quality control
POI: Premature ovarian insufficiency
CAAM: China association for acupuncture and moxibustion
TCM: Traditional Chinese medicine.
